# ^68^Ga-Labeled Fibroblast Activation Protein Inhibitor PET/CT for the Early and Late Prediction of Pathologic Response to Neoadjuvant Chemotherapy in Breast Cancer Patients: A Prospective Study

**DOI:** 10.2967/jnumed.123.266079

**Published:** 2023-12

**Authors:** Ling Chen, Shan Zheng, Linying Chen, Sunwang Xu, Kunlin Wu, Lingjun Kong, Jiajie Xue, Xiangjin Chen, Weibing Miao, Youzhi Zhu

**Affiliations:** 1 Department of Thyroid and Breast Surgery, The First Affiliated Hospital of Fujian Medical University, Fuzhou, China;; 2 Department of Nuclear Medicine, The First Affiliated Hospital of Fujian Medical University, Fuzhou, China;; 3 Department of Pathology, The First Affiliated Hospital of Fujian Medical University, Fuzhou, China; and; 4 Department of Thyroid and Breast Surgery, National Regional Medical Center, Binhai Campus of The First Affiliated Hospital of Fujian Medical University, Fuzhou, China

**Keywords:** fibroblast activation protein inhibitor, ^68^Ga-FAPI, neoadjuvant chemotherapy, pathologic response, breast cancer

## Abstract

^68^Ga-labeled fibroblast activation protein inhibitor (^68^Ga-FAPI) PET/CT has demonstrated promising clinical results, with a higher SUV_max_ and tumor-to-background ratio (TBR) in breast cancer (BC) patients than ^18^F-FDG PET/CT. Here, we aimed to evaluate the suitability of ^68^Ga-FAPI PET/CT for the early and late prediction of the pathologic response to neoadjuvant chemotherapy (NAC) in BC. **Methods:** Twenty-two consecutive patients with newly diagnosed BC and an indication for NAC were prospectively included. All patients underwent standard chemotherapy and ^68^Ga-FAPI PET/CT at baseline, after 2 cycles of NAC (PET2), and 1 wk before surgery (PET3). SUV_max_ was measured in the primary tumor region and positive regional lymph nodes. The expression of fibroblast activation protein in the primary lesion was analyzed by immunohistochemistry. **Results:** Seven patients (31.8%) achieved a pathologic complete response (pCR), and 15 (68.2%) had residual tumors. Thirteen patients (59.1%) showed concentric withdrawal of the primary tumor, and 9 (40.9%) showed diffuse withdrawal. Between PET2 and PET3, the ΔSUV_max_ of the primary tumor (*R*^2^ = 0.822; *P* = 0.001) and metastatic lymph nodes (*R*^2^ = 0.645; *P* = 0.002) were significantly correlated. The absolute values of SUV_max_ and TBR at PET2 and PET3 were lower in patients with pCR than in those without pCR (*P* < 0.05). Moreover, a larger ΔSUV_max_ at any time point was strongly associated with pCR (*P* < 0.05). Similar downward trends in SUV_max_, TBR, and ΔSUV_max_ were observed in the pattern of primary tumor reduction. For predicting pCR, the optimal cutoff values for ΔSUV_max_ after 2 chemotherapy cycles, ΔSUV_max_ before surgery, TBR after 2 chemotherapy cycles, and TBR before surgery of the primary tumor were 3.4 (area under the curve [AUC], 0.890), 1.1 (AUC, 0.978), −63.8% (AUC, 0.879), −90.8% (AUC, 0.978), 7.6 (AUC, 0.848), and 1.4 (AUC, 0.971), respectively. Immunohistochemistry showed that the SUV_max_ and TBR of ^68^Ga-FAPI PET/CT were positively correlated with fibroblast activation protein expression (*P* < 0.001 for both). **Conclusion:** Assessment of early changes in ^68^Ga-FAPI uptake during NAC by ^68^Ga-FAPI PET/CT can predict pCR and primary tumor concentric withdrawal in BC patients. ^68^Ga-FAPI PET/CT has great potential for the early and late prediction of the pathologic response to NAC in BC.

## Visual Abstract

**Figure:**
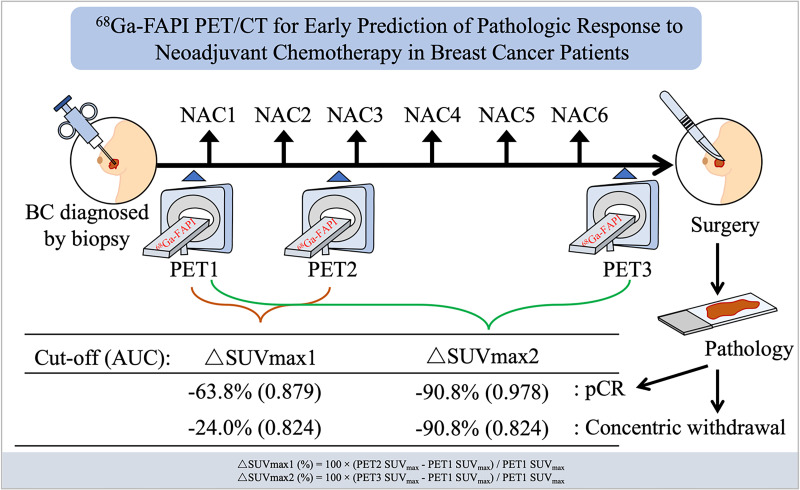

Neoadjuvant therapy has become a very important component of current comprehensive treatments for breast cancer (BC). Neoadjuvant chemotherapy (NAC) can reduce the tumor–node–metastasis stage of BC in most patients to allow surgery or breast preservation to take place; it can also be used to evaluate drug sensitivity in vivo to improve survival (1). A pathologic complete response (pCR) can be used as an alternative research endpoint indicating prognosis in clinical trials of neoadjuvant drugs for BC (2). Whether pCR is achieved is an important basis for adjusting the follow-up adjuvant treatment scheme in BC patients (3*,*^4^).

At present, there is no recognized evaluation method to accurately predict whether pCR can be reached after the full course of treatment at an early stage of neoadjuvant treatment (2 or 4 courses of treatment). Methods involving PET/CT, breast MRI, genomics, and Imageomics (Imageomics Institute) are still in clinical trials. Because the metabolic decline in tumors may occur earlier than the anatomic changes caused by NAC, ^18^F-FDG PET/CT could be an option for assessing changes in tumor viability to monitor the response to NAC (5*,*^6^).

In recent years, ^68^Ga-labeled fibroblast activation protein inhibitor (^68^Ga-FAPI) PET/CT has demonstrated a higher SUV_max_ and tumor-to-background ratio (TBR) than ^18^F-FDG PET/CT in BC (7–9). Furthermore, in the study by Backhaus et al. (10), ^68^Ga-FAPI PET/MRI showed promising diagnostic performance for response assessment after NAC for BC. Therefore, the use of ^68^Ga-FAPI PET/CT to predict the efficacy of NAC for BC would be desirable.

In this prospective study, we first focused on whether ^68^Ga-FAPI PET/CT is useful in predicting pCR in BC patients undergoing NAC. The secondary objectives were to evaluate the changes in ^68^Ga-FAPI PET/CT in the primary tumor and lymph nodes of BC and then assess the withdrawal pattern of BC after NAC. This information is very important in the selection of breast-conserving surgery (BCR) after NAC for BC.

## MATERIALS AND METHODS

### Patients

This prospective study was approved by the Ethics Committee of the First Affiliated Hospital of Fujian Medical University (Approval No. MRCTA; ECFAH of FMU [2019] 293) and as part of an umbrella study registered online at ClinicalTrials.gov (NCT04499365). A total of 25 patients who had newly diagnosed BC confirmed by biopsy and who received NAC were consecutively recruited from March 2021 to July 2022. All subjects signed a written informed consent form.

The key eligibility criteria were as follows: adult patients (18–70 y old); patients who had primary BC confirmed by pathology and who were treatment-naive before ^68^Ga-FAPI PET/CT scans; and patients who met the indications of NAC for BC, completed the full course of NAC, and finally received surgical treatment. Patients who met all of these criteria were included in the study. The exclusion criteria were as follows: patients who were unwilling to undergo a ^68^Ga-FAPI PET/CT scan; patients who could not tolerate chemotherapy or surgery; patients who had any other malignant tumors or who had received any form of antitumor treatment in the past; and patients with evidence of distant metastasis after examination. Three patients were excluded from the study on the basis of these criteria.

All patients underwent ^68^Ga-FAPI PET/CT at baseline (PET1), after 2 cycles of NAC (PET2), and 1 wk before surgery (PET3).

### Examination Procedure for ^68^Ga-FAPI PET/CT

All imaging was performed on dedicated PET/CT scanners (Biograph mCT 64; Siemens Healthcare). The subject was injected with ^68^Ga-FAPI-04 at 1.8–2.2 MBq/kg intravenously into the elbow. After quiet resting for 30 min, a low-dose CT scan without contrast was performed from the top of the skull to the midthigh. CT scanning was performed with the following parameters: 110 kV, 120 mAs, and 3-mm layer thickness. A PET scan was performed immediately after the CT scan. The 3-dimensional mode (matrix, 200 × 200) acquisition method was performed with 3-min/bed positions. Data were obtained by the 3-dimensional row-action maximum-likelihood algorithm method to provide attenuation correction images.

Then, images were analyzed independently on a Syngo MultiModality Workplace (Siemens) by 2 experienced nuclear medicine physicians. Any disagreement was resolved through discussion with a third experienced physician and by integrating other imaging data. If the activity of the primary tumor or regional lymph node exceeded that of the adjacent background tissue, then it was marked as positive. The SUV_max_ was obtained in the region drawn around the primary tumor and positive regional lymph nodes and was calculated for semiquantitative analysis. The TBR was calculated by dividing the lesion SUV_max_ by the SUV_mean_ of the contralateral normal breast tissue. Compared with the baseline value (SUV_max1_), the percentages of ΔSUV_max_ in the region of interest after 2 chemotherapy cycles (SUV_max2_) and before surgery (SUV_max3_) were calculated as follows:

$$\Delta {\text{SUV}}_{\text{max}1}\left(\%\right)=100\times (\text{PET}2{\;\text{SUV}}_{\text{max}}-\text{PET}1{\;\text{SUV}}_{\text{max}})/\;\text{PET}1{\;\text{SUV}}_{\text{max}}$$

and

$$\Delta {\text{SUV}}_{\text{max}2}\left(\%\right)=100\times (\text{PET}3{\;\text{SUV}}_{\text{max}}-\text{PET}1{\;\text{SUV}}_{\text{max}})/\;\text{PET}1{\;\text{SUV}}_{\text{max}}.$$

### NAC Protocols

All patients were confirmed to have BC by a core-needle biopsy. Immunohistochemical staining of estrogen receptor, progesterone receptor, Ki-67, FAP, and human epidermal growth factor receptor (HER2) was performed routinely (supplemental material; supplemental materials are available at http://jnm.snmjournals.org).

Referring to previous studies (11*,*^12^), the BC molecular subtypes were classified as follows: luminal A–like tumors, luminal B/HER2-negative–like tumors, luminal B/HER2-positive–like tumors, HER2-positive–like tumors, and triple-negative–like tumors. The NAC regimen used was the standard recommended by the guidelines (12); details are included in the supplemental material.

### Surgery and Pathology Assessment

Mastectomy was performed in 16 patients, and BCR was performed in 6 patients. According to the initial state of the axillary lymph nodes, 3 patients without clinically suspect signs underwent axillary sentinel lymph node dissection, and 19 patients underwent axillary lymph node dissection.

pCR was defined as the primary breast tumor lacking invasive cancer and the regional lymph node showing a negative result (2). The presence of cancer in situ components was allowed. For non-pCR patients, the residual cancer burden (RCB) scoring system (13) was further used to evaluate the degree of tumor remission. The pattern of tumor reduction (concentric or diffuse) was also observed.

### Statistical Analysis

Statistical analysis was performed using the IBM SPSS 25.0 package (SPSS 25.0 for MAC; IBM SPSS). An independent Student *t* test was used for continuous variables, and the Pearson χ^2^ test or Fisher exact test was used for categoric variables. Receiver operating characteristic curves and the area under the curve (AUC) were determined for regression models according to ^68^Ga-FAPI uptake. The Youden index was used to estimate the optimal cutoff value. Diagnostic agreements between ^68^Ga-FAPI uptake and pCR or primary tumor shrinkage was assessed by sensitivity, specificity, positive predictive value, negative predictive value, and the Cohen κ-index. All statistical analyses were performed using 2-sided tests, and a *P* value of less than 0.05 was considered the threshold for statistical significance.

## RESULTS

### Patient Characteristics

According to the inclusion criteria, a total of 25 patients were enrolled; of these, 3 patients were excluded because they met at least 1 of the exclusion criteria. The clinicopathologic data for the 22 consecutive patients finally enrolled are detailed in Table 1.

**TABLE 1.** Overall Characteristics of Breast Cancer Patients

Characteristic	No. of patients*
Menstrual state	
Premenopausal	12 (54.5)
Postmenopausal	10 (45.5)
Tumor classification	
T2	20 (91.0)
T3	1 (4.5)
T4	1 (4.5)
Lymph node classification	
N0	2 (9.1)
N1	16 (72.7)
N2	2 (9.1)
N3	2 (9.1)
AJCC clinical stage	
IIA	2 (9.1)
IIB	14 (63.6)
IIIA	3 (13.6)
IIIB	2 (9.1)
IIIC	1 (4.5)
Tumor type	
Invasive ductal carcinoma	21 (95.5)
Invasive lobular carcinoma	1 (4.5)
Grade	
G2	8 (36.4)
G3	14 (63.6)
Molecular subtype	
Luminal B/HER2 negative	9 (40.9)
Luminal B/HER2 positive	7 (31.8)
HER2 positive	4 (18.2)
Triple-negative–like	2 (9.1)
Surgery	
BCR	6 (27.3)^†^
Mastectomy	16 (72.7)
Pathologic response	
pCR	7 (31.8)
Non-pCR	15 (68.2)

* Values in parentheses are percentages.

† Of the 6 BCR patients, 2 underwent extensive resection of incisal margin because of diffuse withdrawal in postoperative pathologic evaluation.

AJCC = American Joint Commission on Cancer.

### Correlation Between PET2 and PET3

**FIGURE 1.**
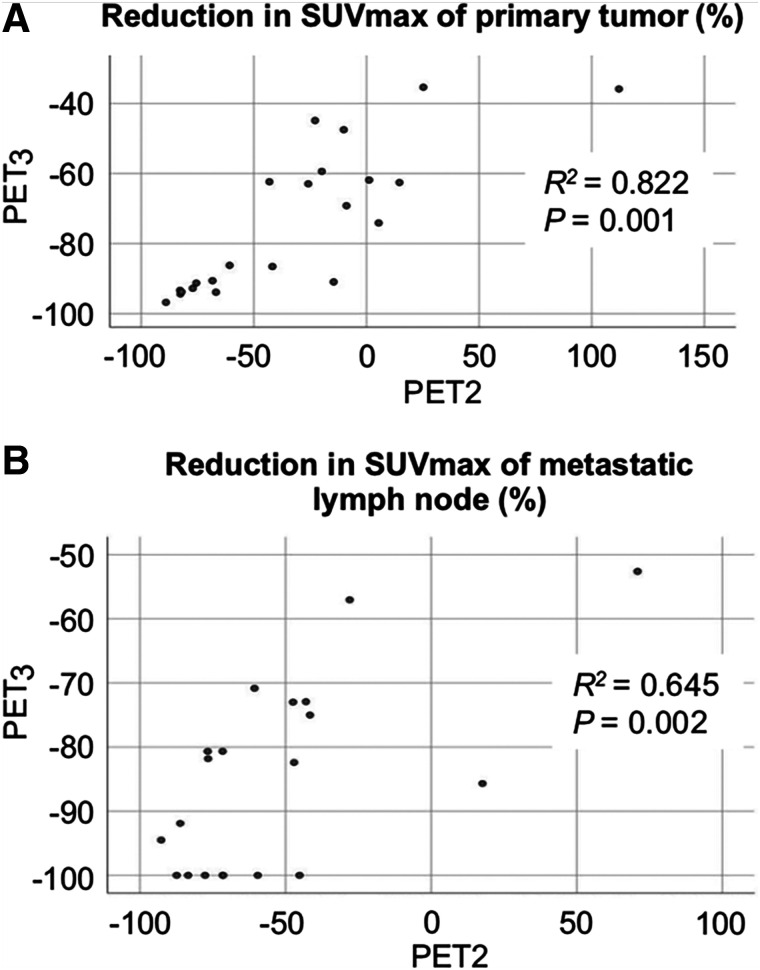
Correlation between relative ΔSUV_max_ of primary tumor (A) and metastatic lymph node (B) at PET2 and PET3.

The median ± SD ΔSUV_max_ of the primary tumor at PET2 was –32.4% ± 48.1% (range, –89.0% to 112.1%), and that at PET3 was –73.9% ± 20.6% (range, –96.8% to –35.4%). In 20 patients with lymph node metastasis, the median ΔSUV_max_ of metastatic lymph nodes (MLNs) at PET2 and PET3 were –54.0% ± 39.0% (range, –92.7% to 70.9%) and –85.0% ± 14.9% (range, –100.0% to –52.6%), respectively. Between PET2 and PET3, there were significant correlations of the ΔSUV_max_ of the primary tumor (*R*^2^ = 0.822 [*P* = 0.001]) and MLNs (*R*^2^ = 0.645 [*P* = 0.002]) (Fig. 1).

### Relationship Between ^68^Ga-FAPI Uptake Changes and pCR

Of the 22 patients, 7 (31.8%) achieved pCR, and 15 (68.2%) showed residual tumor in the final pathology findings. According to the RCB scoring system, there were 7 (31.8%) with RCB0, 1 (4.5%) with RCB1, 6 (27.3%) with RCB2, and 8 (36.4%) with RCB3.

**FIGURE 2.**
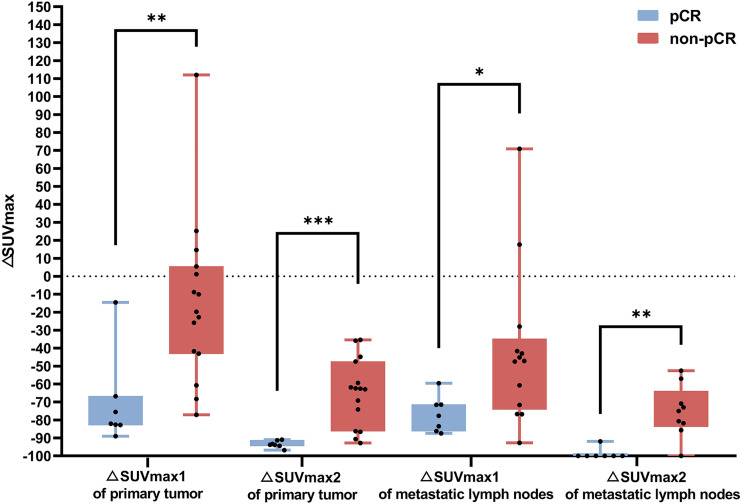
Box plots of ΔSUV_max_ and pathologic response (pCR vs. non-pCR) at PET2 and PET3. *P < 0.05. **P < 0.01. ***P < 0.001.

**FIGURE 3.**
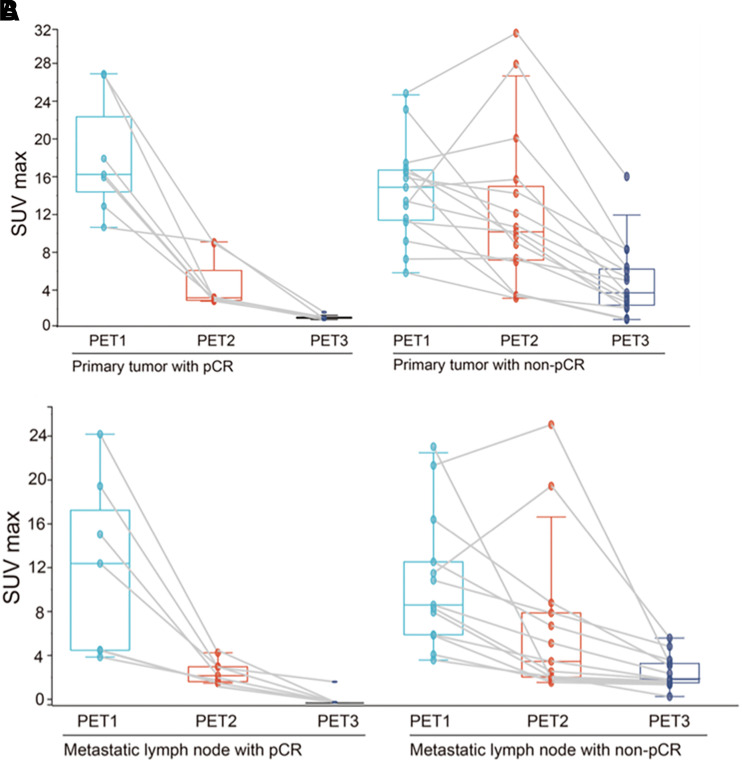
Changes in SUV_max_ of primary tumor (A) and metastatic lymph node (B) according to pathologic response (pCR vs. non-pCR) at PET1, PET2, and PET3.

For the primary tumor, the SUV_max1_ at baseline was not associated with pCR. However, after NAC, most patients showed various degrees of decrease in SUV_max2_, SUV_max3_, TBR after 2 chemotherapy cycles (TBR2), and TBR before surgery (TBR3), with patients with pCR showing a more significant decrease. As Table 2 shows, the absolute values of SUV_max2_, SUV_max3_, TBR2, and TBR3 in patients with pCR were lower than those in patients without pCR (4.7 ± 2.9 vs. 12.7 ± 8.4 [*P* = 0.024], 1.1 ± 0.3 vs. 5.2 ± 3.9 [*P* = 0.001], 4.8 ± 4.0 vs. 11.9 ± 7.4 [*P* = 0.027], and 1.0 ± 0.3 vs. 4.7 ± 3.6 [*P* = 0.001], respectively). Moreover, the larger ΔSUV_max_ between PET1, PET2, and PET3 were strongly associated with the pCR rate (Fig. 2). The ΔSUV_max1_ and ΔSUV_max2_ in the pCR and non-pCR groups were −70.5% ± 25.6% versus –14.6% ± 46.2% (*P* = 0.008) and −93.5% ± 2.0% versus –64.8% ± 18.8% (*P* = 0.001), respectively. Similar downward trends in SUV_max_ and ΔSUV_max_ were also observed in MLNs (Figs. 2 and 3). However, when the ^68^Ga-FAPI uptake of MLNs alone was considered, only SUV_max3_ and ΔSUV_max2_ could better predict pCR than SUV_max2_ and ΔSUV_max1_ (*P* = 0.001).

**TABLE 2.** Correlations Between ^68^Ga-FAPI PET/CT Parameters and Pathologic Response

Parameter	pCR	Non-pCR	*P*	Concentric withdrawal	Diffuse withdrawal	*P*
Primary tumor						
SUV_max1_	18.1 ± 6.4	14.9 ± 5.2	0.224	15.8 ± 6.1	16.1 ± 5.3	0.92
SUV_max2_	4.7 ± 2.9	12.7 ± 8.4	0.024	6.3 ± 3.7	15.8 ± 9.4	0.017
SUV_max3_	1.1 ± 0.3	5.2 ± 3.9	0.001	2.2 ± 1.8	6.4 ± 4.5	0.024
ΔSUV_max1_ (%)	−70.5 ± 25.6	−14.6 ± 46.2	0.008	−53.8 ± 31.1	−1.5 ± 53.0	0.008
ΔSUV_max2_ (%)	−93.5 ± 2.0	−64.8 ± 18.8	0.001	−83.1 ± 15.1	−60.7 ± 20.9	0.008
TBR1	14.7 ± 3.1	15.7 ± 7.9	0.662	15.4 ± 7.0	15.3 ± 7.0	0.974
TBR2	4.8 ± 4.0	11.9 ± 7.4	0.027	6.3 ± 3.8	14.6 ± 8.4	0.02
TBR3	1.0 ± 0.3	4.7 ± 3.6	0.001	1.9 ± 1.3	5.9 ± 4.2	0.022
Metastatic lymph node						
SUV_max1_	11.7 ± 8.1	10.5 ± 6.2	0.723	9.2 ± 6.6	14.2 ± 6.0	0.113
SUV_max2_	2.1 ± 1.0	6.6 ± 7.4	0.054	2.7 ± 1.9	9.3 ± 9.2	0.111
SUV_max3_	0.2 ± 0.6	2.2 ± 1.5	0.001	0.9 ± 1.3	2.7 ± 1.4	0.014
ΔSUV_max1_ (%)	−76.7 ± 10.0	−41.7 ± 43.6	0.053	−64.0 ± 19.4	−36.3 ± 58.7	0.25
ΔSUV_max2_ (%)	−98.8 ± 3.1	−77.5 ± 13.1	0.001	−88.3 ± 15.1	−78.7 ± 13.2	0.171

TBR1 = TBR at baseline.

### Relationship Between ^68^Ga-FAPI Uptake Changes and Primary Tumor Withdrawal

With regard to the pattern of primary tumor reduction, there were 13 patients (59.1%) with concentric withdrawal and 9 (40.9%) with diffuse withdrawal. Of the 6 BCR patients, 2 underwent extensive resection of the incisal margin because of diffuse withdrawal in the postoperative pathologic evaluation.

**FIGURE 4.**
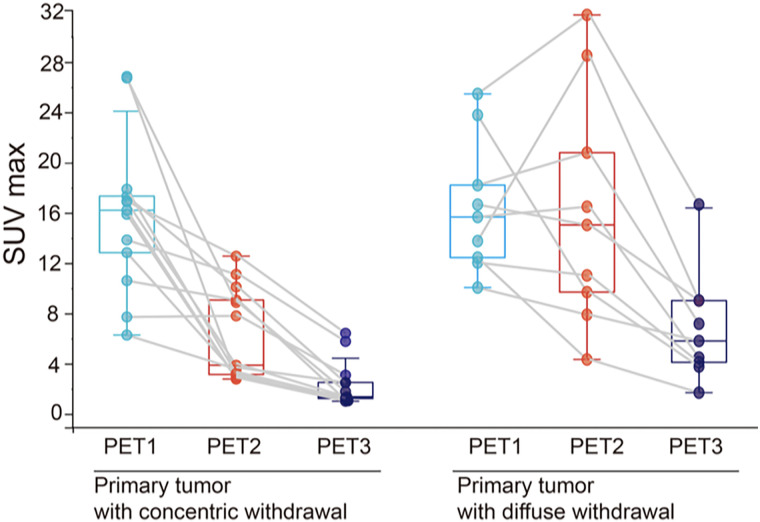
Changes in SUV_max_ of primary tumor according to pathologic response (concentric withdrawal vs. diffuse withdrawal) at PET1, PET2, and PET3.

Compared with those in patients with diffuse withdrawal, the SUV_max2_, SUV_max3_, TBR2, and TBR3 of the primary tumor in patients with concentric withdrawal were lower, and the decline in ΔSUV_max1_ and ΔSUV_max2_ was greater (all *P*s < 0.05). Regarding the ^68^Ga-FAPI uptake changes in MLNs, there was little correlation with the regression pattern of the tumor (Fig. 4; Table 2).

### Prediction of pCR and Concentric Withdrawal According to ^68^Ga-FAPI Uptake

In predicting pCR, 3.4 (AUC, 0.890), 1.1 (AUC, 0.978), 7.6 (AUC, 0.848), and 1.4 (AUC, 0.971) were the optimal cutoff values for SUV_max2_, SUV_max3_, TBR2, and TBR3 of the primary tumor, respectively. The optimal cutoff values for ΔSUV_max1_ and ΔSUV_max2_ were −63.8% (AUC, 0.879) and −90.8% (AUC, 0.978), respectively. The optimal cutoff values for SUV_max3_ and ΔSUV_max2_ of the MLNs were 0.5 (AUC, 0.901) and −88.8% (AUC, 0.945), respectively.

In predicting concentric withdrawal of the primary tumor, the optimal cutoff values for SUV_max2_, SUV_max3_, TBR2, and TBR3 of the primary tumor were 14.3 (AUC, 0.824), 3.1 (AUC, 0.857), 9.5 (AUC, 0.821), and 2.5 (AUC, 0.872), respectively. Those for ΔSUV_max1_ and ΔSUV_max2_ were −24.0% (AUC, 0.824) and −90.8% (AUC, 0.824), respectively.

These selected critical values for primary tumor uptake were further used as cutoff values to evaluate the diagnostic agreement (Table 3). The SUV_max2_ and TBR3 of the primary tumor criteria showed the highest sensitivity in predicting pCR (100% for all). The SUV_max3_ and ΔSUV_max2_ of the primary tumor criteria had the highest specificity in predicting pCR (100% for all). The SUV_max3_, TBR3, and ΔSUV_max2_ of the primary tumor criteria had the highest κ-index in predicting pCR (0.899).

**TABLE 3.** Diagnostic Agreement of ^68^Ga-FAPI Uptake Cutoff Values with Favorable Pathologic Response of Primary Tumor

Variable	pCR					Concentric withdrawal				
	Sensitivity (%)	Specificity (%)	PPV (%)	NPV (%)	κ-index	Sensitivity (%)	Specificity (%)	PPV (%)	NPV (%)	κ-index
ΔSUV_max1_	75	92.9	85.7	86.7	0.697	83.3	70	76.9	77.8	0.538
SUV_max2_	100	88.2	71.4	100	0.773	76.5	100	100	55.6	0.596
ΔSUV_max2_	87.5	100	100	93.3	0.899	100	64.3	61.5	100	0.567
SUV_max3_	87.5	100	100	93.3	0.899	91.7	80	84.6	88.9	0.723
TBR2	85.7	80	66.7	92.3	0.611	76.9	77.8	83.3	70	0.538
TBR3	100	93.3	85.7	100	0.899	76.9	88.9	90.9	72.7	0.636

PPV = positive predictive value; NPV = negative predictive value.

In predicting primary tumor concentric withdrawal, the ΔSUV_max2_ of the primary tumor criteria had the highest sensitivity (100%). The SUV_max2_ of the primary tumor criteria had the highest specificity (100%). The SUV_max3_ of the primary tumor criteria had the highest κ-index (0.723).

### Association Between FAP Immunohistochemistry and ^68^Ga-FAPI Uptake

**FIGURE 5.**
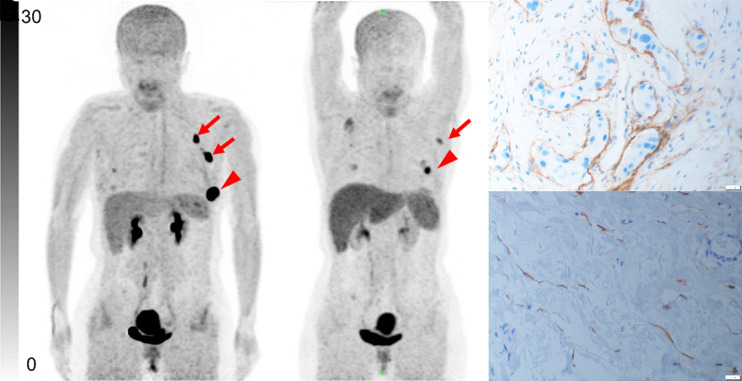
Representative ^68^Ga-FAPI PET/CT images of 48-y-old woman who had left invasive ductal carcinoma and who achieved pCR. (A) Baseline ^68^Ga-FAPI PET/CT maximum-intensity-projection (MIP) image demonstrated primary lesion (red arrowhead; SUV_max_, 26.72) and ^68^Ga-FAPI–avid lymph node in left axilla (red arrows). (B) ^68^Ga-FAPI PET/CT MIP image after 2 cycles of NAC showed that primary lesion (red arrowhead; SUV_max_, 8.87) and axillary lymph nodes (red arrow) were reduced in size and radioactivity uptake. (C and D) Representative images of immunohistochemical staining for FAP before NAC showed strong FAP-positive staining in stromal cells (C), which was significantly decreased on tumor bed after 6 cycles of NAC (D).

**FIGURE 6.**
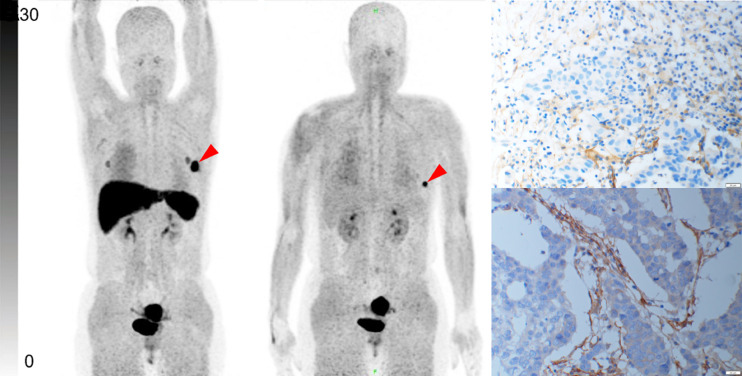
Representative ^68^Ga-FAPI PET/CT images of 46-y-old woman who had left invasive ductal carcinoma and who did not achieve pCR. (A) Baseline ^68^Ga-FAPI PET/CT maximum-intensity-projection (MIP) image demonstrated primary lesion (red arrowhead; SUV_max_, 11.62). (B) ^68^Ga-FAPI PET/CT MIP image after 2 cycles of NAC showed that primary lesion (red arrowhead; SUV_max_, 10.59) was smaller and that radioactivity uptake was slightly lower than before. (C and D) Representative images of immunohistochemical staining for FAP before NAC showed strong FAP-positive staining in stromal cells (C), but no obvious change was observed on tumor bed after 6 cycles of NAC (D).

We performed immunohistochemical staining for FAP on both biopsy specimens and postoperative specimens of the primary tumor. The SUV_max_ and TBR of ^68^Ga-FAPI PET/CT were positively correlated with FAP expression in the stroma of the BC lesions (*r* = 0.886 [*P* < 0.001] and *r* = 0.556 [*P* < 0.001], respectively). Figures 5 and 6 show images of 2 patients as representative examples.

## DISCUSSION

In the present study, we demonstrated the clinical practicality of using ^68^Ga-FAPI uptake based on preprocessed ^68^Ga-FAPI PET/CT for predicting the pathologic response to NAC treatment in BC patients.

Under NAC, the lower SUV_max2_, SUV_max3_, TBR2, and TBR3 as seen on ^68^Ga-FAPI PET/CT and the larger ΔSUV_max1_ and ΔSUV_max2_ were strongly associated with a higher pCR rate. Moreover, ^68^Ga-FAPI PET/CT had a high value in the early response evaluation. Patients with no response at PET2 had a higher rate of obtaining a score of RCB2 or RCB3 in the final pathologic evaluation. Our research showed that ^68^Ga-FAPI PET/CT can rapidly provide feedback on tumor changes through ^68^Ga-FAPI uptake after 2 cycles of chemotherapy. Accurate evaluation can lead clinicians to adjust their chemotherapy regimen as soon as possible to avoid ineffective chemotherapy for patients who have not achieved pCR or who show poor therapeutic effects.

In recent years, ^18^F-FDG PET/CT has been optionally used to monitor the responses to NAC in BC patients (5*,*^14^). In previous metaanalyses for ^18^F-FDG PET/CT, it was found that the predictive value of SUV_max_ has high sensitivity but relatively low specificity in predicting pCR (15*,*^16^). There are 2 obvious limitations in the clinical application of ^18^F-FDG PET/CT for the early prediction of a pathologic response. First, ^18^F-FDG PET has limited sensitivity in detecting small breast lesions, lobular cancer, or low-grade BC (17*,*^18^). A previous study showed that ΔSUV_max_ obtained with ^18^F-FDG PET can only be evaluated when the TBR is higher than 5 in BC (19). In addition, the patient’s blood glucose concentration, insulin concentration, and level of inflammation may influence ^18^F-FDG PET/CT parameters (20). During NAC, insulin resistance and blood sugar changes may occur, and myelosuppression can also cause inflammation. These factors also increase the instability of ^18^F-FDG PET/CT monitoring.

Reportedly, the expression of FAP is enhanced at tumor boundaries and invasive frontiers, such as tumor cell protrusions, under the microscope (21). Ristau et al. (22) reported the complementary role of ^68^Ga-FAPI PET/CT in accurately depicting the volume of primary tumors. Their results indicated that ^68^Ga-FAPI PET/CT examination is less affected by the patient’s blood sugar and inflammatory factors. In addition, in our previous research, ^68^Ga-FAPI PET/CT showed a higher SUV_max_ and TBR than ^18^F-FDG PET/CT in primary tumors, and it also showed advantages in identifying regional lymph node metastasis (9). In addition, previous studies found no differences in ^68^Ga-FAPI uptake based on histopathologic features such as immunohistochemistry and grading (7*,*^8^). These factors all facilitate precise evaluation using ^68^Ga-FAPI PET/CT. In the study by Backhaus et al. (10), integrated ^68^Ga-FAPI PET/MRI was used to classify the response in BC correctly on the basis of readers’ visual assessment or TBR. In the present study, ^68^Ga-FAPI PET/CT measures also showed high accuracy in predicting pCR.

In the present study, we also explored the ability of ^68^Ga-FAPI PET/CT to predict tumor withdrawal patterns after NAC. Downstaging primary tumors to allow for BCR is one of the goals of NAC. A metaanalysis of the Early Breast Cancer Trialists’ Collaborative Group showed that NAC can increase the probability of BCR, but it can also lead to an increase in local recurrence rate (23). How to accurately evaluate residual tumors after NAC is the crux of clinical treatment. Currently, MRI is the most commonly used method for evaluating tumor withdrawal after NAC. In the present study, the parameters of FAP uptake better evaluated the tumor withdrawal pattern, especially when ^68^Ga-FAPI PET/CT was done 1 wk before surgery.

The present study has some limitations that must be considered. First, this pilot prospective study was conducted in a single institution with a limited sample size. Further multiinstitutional studies with large samples are needed to validate our results. Second, we did not explore those BC patients separately on the basis of molecular subtypes. BC is a heterogeneous disease, and different molecular subtypes and chemotherapy regimens can also affect the reduction in SUVs (24*,*^25^). In the present study, we did not include patients with luminal A subtype BC. A larger patient population is needed in further trials to clarify this point. The applicability of ^68^Ga-FAPI PET/CT to predict the early pathologic response of different subgroups of BC is the focus of our follow-up study. Third, in the present study, neoadjuvant treatment only included chemotherapy (with or without HER2-targeted therapy). Therefore, the ability of ^68^Ga-FAPI PET/CT to assess the response to other neoadjuvant treatments, such as hormonal therapy or immunotherapy, cannot be inferred.

## CONCLUSION

The present study suggests that during NAC, early changes in ^68^Ga-FAPI uptake (SUV_max_, TBR, and ΔSUV_max_) assessed by ^68^Ga-FAPI PET/CT are powerful parameters for predicting pCR in BC patients. ^68^Ga-FAPI PET/CT parameters can rapidly provide feedback on tumor changes after only 2 cycles of chemotherapy. For predicting primary tumor concentric withdrawal and determining the appropriateness of BCR, imaging at PET3 is further recommended. Future studies should consider cancer subtypes and chemotherapy regimens and further validate ^68^Ga-FAPI PET/CT as an early response indicator to support its use as a better clinical tool for guiding patient treatment.

## DISCLOSURE

This research was funded by the Fujian Provincial Health Technology Project (2022CXA020) and the Natural Science Foundation of Fujian Province (2021J01720). No other potential conflict of interest relevant to this article was reported.

## Notes

KEY POINTS **QUESTION:** Can ^68^Ga-FAPI PET/CT predict the pathologic response to NAC in BC patients? **PERTINENT FINDINGS:** Assessment of early changes in ^68^Ga-FAPI uptake (SUV_max_, TBR, and ΔSUV_max_) during NAC by ^68^Ga-FAPI PET/CT can predict pCR and primary tumor concentric withdrawal in BC patients. **IMPLICATIONS FOR PATIENT CARE:** ^68^Ga-FAPI PET/CT has great potential for the early prediction of a pathologic response to NAC in BC, supporting its use as a better clinical tool for guiding patient treatment.
